# Evaluation of post-operative surveillance strategies for esophageal and gastric cancers: a systematic review and meta-analysis

**DOI:** 10.1093/dote/doac034

**Published:** 2022-07-05

**Authors:** Swathikan Chidambaram, Viknesh Sounderajah, Nick Maynard, Sheraz R Markar

**Affiliations:** Department of Surgery and Cancer, Imperial College London, London, UK; Department of Surgery and Cancer, Imperial College London, London, UK; Department of Upper GI Surgery, Churchill Hospital, Oxford University Hospitals, Oxford, UK; Department of Surgery and Cancer, Imperial College London, London, UK; Department of Molecular Medicine and Surgery, Karolinska Institute, Stockholm, Sweden; Department of Upper GI Surgery, Churchill Hospital, Oxford University Hospitals, Oxford, UK

**Keywords:** esophageal adenocarcinoma, esophageal and gastric cancer, esophageal and gastric surgery, surveillance

## Abstract

**Background:**

There is no consensus or guidelines internationally to inform clinicians of how patients should be monitored for recurrence after esophagogastric resections.

**Aim:**

This systematic review and meta-analysis summarizes the latest evidence investigating the usefulness of surveillance protocols in patients who underwent esophagectomy or gastrectomy.

**Methods:**

A systematic review of the literature was performed using MEDLINE, EMBASE, the Cochrane Review and Scopus databases. Articles were evaluated for the use of surveillance strategies including history-taking, physical examination, imaging modalities and endoscopy for monitoring patients post-gastrectomy or esophagectomy. Studies that compared surveillance strategies and reported detection of recurrence and post-recurrence survival were also included in the meta-analysis.

**Results:**

Fifteen studies that described a surveillance protocol for post-operative patients were included in the review. Seven studies were used in the meta-analysis. Random-effects analysis demonstrated a statistically significant higher post-recurrence survival (standardized mean difference [SMD] 14.15, 95% CI 1.40–27.26, *p* = 0.03) with imaging-based planned surveillance post-esophagectomy. However, the detection of recurrence (OR 1.76, 95% CI 0.78–3.97, *p* = 0.17) for esophageal cancers as well as detection of recurrence (OR 0.73, 95% CI 0.11–5.12, *p* = 0.76) and post-recurrence survival (SMD 6.42, 95% CI –2.16–18.42, *p* = 0.14) for gastric cancers were not significantly different with planned surveillance.

**Conclusion:**

There is no consensus on whether surveillance carries prognostic survival benefit or how surveillance should be carried out. Surveillance may carry prognostic benefit for patients who underwent surgery for esophageal cancer. Randomized controlled trials are required to evaluate the survival benefits of intensive surveillance strategies, determine the ideal surveillance protocol and tailor it to the appropriate population.

## INTRODUCTION

Gastric and esophageal cancers are aggressive and associated with a 5-year overall survival rate of approximately 25%.^1^^,^^2^ This poor prognosis can be primarily attributed to a recurrence rate as high as 80% even after multi-modal treatment with curative intent.^3–6^ Routine post-operative surveillance of patients can help to detect recurrence and initiate intervention early. However, there is no international consensus on the best strategy for follow-up of patients who underwent surgery with curative intent for esophago-gastric cancers.^7^ Surveillance has been beneficial in detecting recurrence in patients who underwent surgical resection for colorectal cancer, leading to improved survival outcomes. There is well-established evidence that have demonstrated an overall survival advantage from intensive surveillance in patients with colorectal cancer.^8^^,^^9^ Subsequently, surveillance protocols are now standard in various international guidelines.^10^^,^^11^ In contrast, the evidence for routine intensive surveillance for esophago-gastric cancers is ambiguous. Some studies have reported success in detecting asymptomatic recurrences earlier than symptomatic recurrences when using an intense post-operative surveillance program. Other studies noted that the overall survival was not increased in these studies, especially when factoring in lead-time biases.^12^

Currently, there is no randomized controlled trial that has investigated the impact of intensive routine surveillance against symptom-triggered surveillance. As a result, there is subsequently no internationally accepted surveillance strategy or regime for patients after surgical resection. Messager *et al*.^13^ reported a wide geographical variation in surveillance strategies and a lack of consensus between the guidelines even within a relatively homogeneous group of countries in western and central Europe. The most recent review on this topic was in 2012, and since then, more work has been carried out to determine if intensive surveillance has a prognostic benefit, and more importantly, the structure of the ideal regimen.^14^ Furthermore, routine surveillance was traditionally not advocated given that the treatments for recurrent esophago-gastric cancers were limited. The recent advances in the radical management of recurrent cancers with multimodal therapy (surgery and/or chemoradiotherapy) provides further justification for adopting a more intensive approach towards post-operative surveillance. This systematic review and meta-analysis aims to summarize the latest evidence investigating the usefulness of various surveillance protocols in patients who underwent surgical intervention for esophago-gastric cancers.

## METHODS

A systematic review of studies evaluating recurrence and survival in patients who were followed-up after surgery for esophageal and gastric cancers was conducted. This systematic review follows the Preferred Reporting Items for Systematic Reviews and Meta-Analyses (PRISMA) guidelines.^15^ Studies that compared planned and unplanned surveillance strategies were subsequently included in the meta-analysis.

### Search strategy

A literature search was performed in MEDLINE, EMBASE, Cochrane Central Register of Controlled Trials, and CINAHL databases on the 17 July 2021 to identify relevant studies describing surveillance strategies in patients who have undergone surgery for esophageal and gastric cancers. The search included the following keywords ‘esophageal cancer,’ ‘esophageal adenocarcinoma,’ ‘esophageal squamous cell carcinoma,’ ‘esophagectomy,’ ‘Ivor Lewis,’ ‘McKeown,’ ‘trans-hiatal esophagectomy,’ ‘gastric cancer’ OR ‘gastric adenocarcinoma’ OR ‘gastric squamous cell carcinoma’; ‘gastrectomy’ OR ‘total gastrectomy’ OR ‘partial gastrectomy’ OR ‘subtotal gastrectomy’ OR ‘distal gastrectomy’; and ‘surveillance’ OR ‘monitoring’ OR ‘follow-up.’ The individual strings were combined using the AND modifier. References of included articles were screened and a hand-search was performed to identify missing articles. Two reviewers (SC and VS) independently assessed the titles and abstracts for inclusion of relevant references. In cases where there was disagreement for inclusion, a third author (SRM) was consulted.

### Study selection

Randomized controlled trials (RCTs), quasi-randomized trials, cohort studies and case–control studies were included if they investigated the use of a surveillance protocol in patients who had undergone surgery for gastric cancer or oesophageal cancer, or compared the factors between symptomatic and asymptomatic recurrence. Any form of surveillance such as regular clinical follow-up (history and examination), blood tests, radiological investigations and endoscopy were considered as part of a surveillance strategy. Any type of surgery carried out with a curative intent regardless of operative technique, approach and additional procedures was included. Owing to the heterogeneity of cases included, it was not feasible to control for a specific grade, stage or histological sub-type of gastric cancer, hence all cancers of the stomach were included. Studies involving patients who underwent surgery alongside adjuvant or neoadjuvant chemotherapy were included as well.

Review articles and case reports were excluded. Studies were also excluded if there was no comparative group present. Comparative studies were excluded if no outcome data was provided for the control or the intervention group. Studies were excluded if surveillance post-surgery was performed in a cohort of patients who would have required monitoring regardless, such as patients with hereditary syndromes (CHD1 mutation leading to hereditary diffuse gastric cancer). Finally, articles were excluded if patients did not undergo surgery but underwent only other curative therapies and if other cancer types were included.

### Outcome measures and data extraction

Our main aim was to assess the impact of post-operative surveillance on survival outcomes, hence primary outcome measures were length of survival and detection of recurrence. Secondary aims included readmissions as well as trigger of other investigations or interventions (e.g. imaging shows evidence of recurrence hence endoscopy was carried out); and the incidence of postoperative morbidity assessed by a Clavien-Dindo Classification (CDC) of 2 or higher. In addition, the following data were extracted from each study: first author, year of publication, study design, sample size, demographic data (age and gender), oncological details (stage, grade and histological subtype of cancer); surgical intervention (operation, approach, intervention, details of lymphadenectomy); other forms of treatment undergone (adjuvant and/or neoadjuvant chemotherapy and/or radiotherapy) and details of surveillance (frequency, setting, persons involved, clinical evaluation carried out, investigations undergone and why these were performed). The definition of recurrence was based on the primary studies, which used a combination of clinical symptoms, CT or PET evidence, and direct visualization on endoscopy. However, given that the included studies investigated the use of CT or PET, detection of recurrence was based primarily on evidence from imaging.

### Quality assessment of selected studies

Two reviewers (SC and VS) assessed quality of each included study by independently evaluating the risk of bias using the Newcastle-Ottawa Scale (NOS) for the assessment of non-randomized studies.^16^^,^^17^ The NOS scores ranging from 0 to 9, with a higher score indicating a lower risk of bias. In this review, we considered a score of 0–3, 4–6 and 7–9 as low, moderate and high quality of studies, respectively.

### Statistical methods

Review Manager 5.3 (Cochrane Collaboration, Oxford, United Kingdom) was used for statistical analysis of the data. Two types of modeling were used to assess the heterogeneity of the data: fixed-effects and random-effects. The random-effects model was chosen for all analysis due to the significant heterogeneity between studies. Data are given as odds ratio and 95% confidence intervals (CI) for all non-continuous data, and as standardized mean difference and 95% CI for all continuous data. In all cases, statistical heterogeneity was assessed by using *I*^2^ statistic and was categorized as low, moderate and high for an *I*^2^ statistic of above 25%, 50% and 75%, respectively. Results above 60% were considered as substantial heterogeneity. All data given as medians were converted to means and standard deviations, as outlined by Hozo *et al*.^18^

## RESULTS

### Study selection

**Fig. 1:**
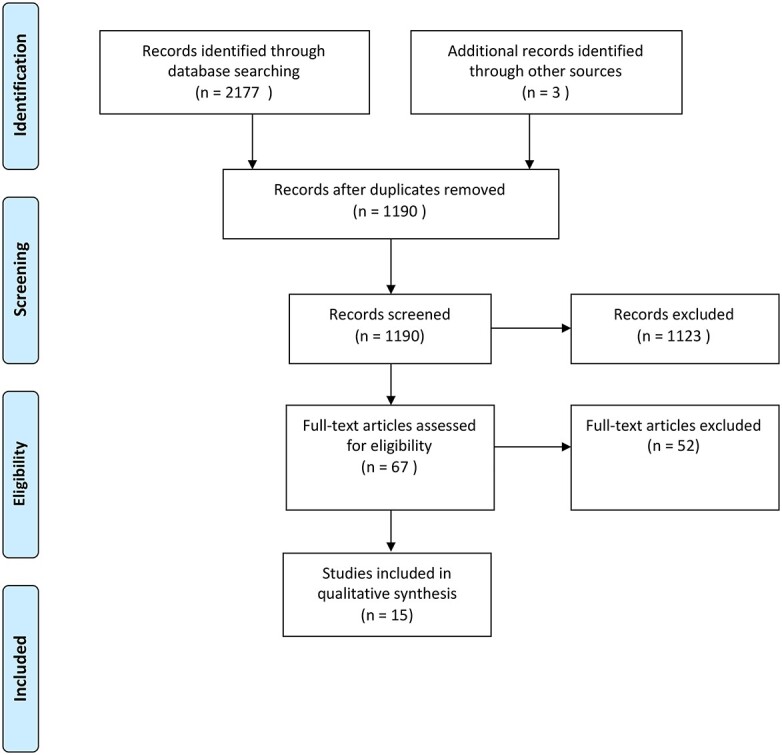
PRIMSA diagram of studies included.

The database search yielded a total of 2180 studies. After duplicated were removed, titles and abstracts of the remaining 1190 studies were assessed for eligibility, and 1123 studies were removed. A further 52 studies were excluded after full-text review due to incompatible outcome measures or study design (Figure 1).^15^ Fifteen studies that described a surveillance program for following up patients post-gastrectomy or esophagectomy were included (Table 2).^6^^,^^12^^,^^19–31^

### Quality appraisal

Assessment of studies using the Newcastle-Ottawa tool showed that studies were of a moderate-high quality (Table 1). The non-randomized studies included were evaluated for sources of bias using the NOS. All studies achieved an excellent score of 7/8 on the NOS. They attained maximum points for the ‘selection’ category. Some risk of bias was present due to heterogeneity of the population that reduced the comparability of the study cohorts. Two studies did not report an accurate follow-up time.

**Table 1:** Newcastle-Ottawa quality assessment scale for cohort studies

Author (year)	Selection	Comparability	Outcome
Bilici	***	**	**
Hosokawa	****	**	**
Lee	***	**	***
Eom	***	*	***
Kodera	***	**	**
Park	***	*	**
Peixoto	****	**	**
Tan	***	*	**
Antonowicz	****	**	**
Bennett	****	**	**
Jiang	****	**	**
Abate	***	**	***
Lou	****	**	**
Elliott	***	**	***
DeSouza	***	**	***

### Study and patient characteristics

Fifteen different studies were included in this paper. In total, this accounted for 6271 patients. Patients underwent a combination of partial gastrectomy, subtotal gastrectomy or total gastrectomy for gastric cancers; one study did not explicit state the type of gastrectomy carried out.^27^ One study included only patients with early gastric cancer, while other studies included patients with tumors of stages T1–T4 (Table 2). Accordingly, except 1 study, all other patients underwent chemotherapy and/or radiotherapy. Histological subtypes as reported in six of the studies included poor to well-differentiated carcinoma of adeno, squamous or signet ring morphologies. Studies on esophageal cancer included T1–T4 cancers in most studies of both adenocarcinoma and squamous cell histological subtypes. A range of procedures including transthoracic, trans-hiatal, vagal-sparing or minimally invasive thoracoscopic/laparoscopic approaches were used. Some studies also carried out gastrectomy due to tumor location. All of the studies involved a combination of surgery and chemoradiotherapy as per standardized guidelines (Table 3).

**Table 2:** Characteristics of patients

Author	Year	Design	Sample size	Age	Sex (males)	Stage	Cancer type	Histological subtype	Operation	Other forms of treatment
Bennett	2005	Retrospective	283 99	64 63	267 115	T1–2 133 T3–4 249	Gastric	Well/mod diff 109 Poor diff 160	R0 gastrectomy	Nil (patients were excluded)
Bilici	2013	Prospective	173		118	TI 5 T2 41 T3 102 T4 25	Gastric	Well differentiated 7 Mod differentiated 79 Poorly differentiated 97	D1 or D2 gastrectomy	140 received adjuvant chemoradiotherapy
Hosokawa	2002	Prospective	642	—	—	Early gastric cancer	Gastric	Adenocarcinoma	Partial gastrectomy	
Lee	2005	Retrospective	834	56 52	135 404	TI 330 T2 314 T3 221 T4 115	Gastric	Well differentiated 63 Mod differentiated 205 Poorly differentiated 179	Total gastrectomy	
Eom	2011	Retrospective	310	56 57	45 162	TI 10 T2 89 T3 184 T4 27	Gastric	Differentiated adenocarcinoma 73 Undifferentiated adenocarcinoma or signet ring cell carcinoma 233		Adjuvant chemotherapy
Kodera	2003	Retrospective	197	62 60	59 74	TI 20 T2 55 T3 63 T4 59	Gastric	N/A	Total gastrectomy 95	Adjuvant chemotherapy 100
Park	2016	Prospective	2785	57	1825	T1A 1340 T1B 290 T2A 217 T2B 244 T3A 181 T3B 211 T3C 302	Gastric	Adenocarcinoma Squamous cell carcinoma	Subtotal gastrectomy (2028) Total gastrectomy (757)	N/A
Peixoto	2014	Prospective	292	63	223	T0/T1 25 T2 81 T3 126 T4 16 Unreported 44	Gastric	230 adenocarcinoma 36 squamous cell carcinoma 26 signet ring cell carcinoma	Open gastrectomy	1. Neoadjuvant or adjuvant combined modality chemoradiotherapy (65%) 2. Definitive chemoradiotherapy (23%) 3. Perioperative chemotherapy (12%)
Tan	2007	Prospective	102	57.8 61.9	30 28	TI 23 T2 23 T3 50 T4 6	Gastric	N/A	64 subtotal gastrectomy 38 total gastrectomy	36 adjuvant therapy. This consisted of either chemotherapy or radiotherapy or both.
Abate	2009	Retrospective	174	56 69	27	T1 T2 T3 T4	Esophageal	Adenocarcinoma only	Transthoracic en bloc, transhiatal, vagal-sparing, or minimally invasive thoracoscopic/laparoscopic esophagectomy	47 had neoadjuvant therapy
Antonowicz	2015	Retrospective	17 44	61 64	16 38	T1 5 T2 13 T3 41 T4 1	Esophageal	50 adenocarcinoma 7 squamous cell carcinoma	Open: 13 Minimally invasive: 45 Transhiatal: 3	Neoadjuvant chemotherapy: 41 patients (67.4%) of cohort
Jiang	2020	Retrospective	210	64.1	153	T1 15 T2 35 T3 47 T4 113	Gastric and Esophageal		Esophagectomy 25 Gastrectomy 99 Esophago-gastrectomy 86	Surgery plus adjunctive therapy 150 Neoadjuvant chemotherapy only 19 Adjuvant chemotherapy only 11 Neoadjuvant and adjuvant chemotherapy 25 Neoadjuvant chemoradiation only 56 Adjuvant chemoradiation only 29 Neoadjuvant chemotherapy and adjuvant chemoradiation 7 Neoadjuvant chemoradiation and adjuvant chemotherapy 3
Lou	2013	Retrospective	1147			T1 366 T2 343 T3 284	Esophageal	942 adenocarcinoma 205 squamous cell carcinoma	Esophagectomy	723 had neoadjuvant chemotherapy
Elliott	2020	Prospective	4682			—	Esophageal		Esophagectomy	60% chemoradiotherapy
DeSouza	2018	Retrospective	273		230	—	Esophageal	240 adenocarcinoma	Esophagectomy	206 chemotherapy 196 radiotherapy

**Table 3:** Characteristic of surveillance protocol and outcome measures

Author	Surveillance protocol	Follow-up (months)	Definition of recurrence	Recurrence	Survival	Conclusion
Bennett	As per National Comprehensive Cancer Network guidelines		Confirmed by pathological findings	382 patients	PRS: 13.5 months for asymptomatic patients 4.8 months for symptomatic patients Disease specific survival: 29.4 months if asymptomatic vs 21.6 months if symptomatic	Follow up did not identify asymptomatic recurrence earlier than symptomatic recurrence.
Bilici	History and exam every 3 month in first year, every 6 months in the second year, and annually for at least 5 years CXR and CT every 3 months in first year, every 6 months in 2nd year, and annually thereafter for 5 years. Annual OGD	Median 22.5			OS: 18.3 months in asymptomatic 12.3 months in symptomatic Disease-free survival: 11.1 versus 9.3 months PRS: 4.9 versus 3.1 months	Symptomatic recurrence is an important prognostic factor for PRS of patients with gastric cancer after a curative gastrectomy
Lee	Examination, CXR, CT and OGD at 6 to 12 months	36		233	—	Follow-up endoscopy after total gastrectomy for gastric cancer is useful in detecting complications and tumor recurrence.
Hosokawa	Surveillance endoscopy examinations at short intervals - annually if possible and biennially at the longest.	60		15	—	Periodical surveillance endoscopy for gastric remnant cancer is recommended after surgery for early gastric cancer.
Eom	Advanced: bloods every 3 m for 3 years CXR, CT, OGD, and tumor marker 6 monthly for first 3 years and annually for the next 2 years. Annual OGD annually for 5 years Early: Bloods at 3 m and 6 m. CXR, CT, OGD, and tumor marker annually for 5 years	60			PRS: 4.9 months in the undetected group vs. 13.0 months in the detected group OS: No significant difference between the 2 cohorts (based on Kaplan–Meier curves)	Oncologic effectiveness of regular follow-up after curative was unsatisfactory
Kodera	History, examination, blood tests every 3 months for the first postoperative year and every 6 months for at least 5 years. USS or CT every 6 months. Endoscopy annually beginning 1 to 1.5 years after surgery.	60		Asymptomatic Recurrence (88) Symptomatic Recurrence (109)	Better PRS if detected in asymptomatic patients, but no difference in overall survival.	Early detection of asymptomatic gastric cancer recurrence did not improve overall survival of patients with recurrence after curative resection.
Park	1. <3 months 2. 3–6 months 3. 6–12 months CT, PET and OGD at the respective time points	12		376 (13.5%)	PRS: <3 m: HR 0.954 3–6 m: HR 0.994, OS: <3 m: HR 0.969 3–6 m: HR 0.955 6–12 m: HR 1.00	Asymptomatic patients should not undergo surveillance with imaging studies more than once a year.
Peixoto	Cohort 1: discharge to primary care (89) Cohort 2: follow-up with oncologist (18) Cohort 3: specialist follow-up with lab tests (32) Cohort 4: specialist follow-up with imaging or endoscopy (153)	36		Cohort 1: HR 1.0 Cohort 2: HR 0.73 Cohort 3: HR 1.77 Cohort 4: HR 1.09	Cohort 1: HR 1.0 Cohort 2: HR 0.47 Cohort 3: HR 1.67 Cohort 4: HR 0.78	Outcomes were comparable irrespective of surveillance strategy. Intensive follow-up with routine imaging and endoscopy may not be justified.
Tan	Intensive regimen: physical examination, tumor markers assays, CT scan >1 every 12 months Regular regimen: history taking, physical examination with tumor marker assay and CT scan of not >1 per year	60		49% in intensive group 43% in the regular group Recurrences detected earlier in intensive group (11.5 vs. 19.2 m)	5-year survival rate 43% in the intensive regimen, 34% in the regular group. OS: 4.1 years in the intensive group; 3.8 years in the regular group	Intensive follow-up resulted in the earlier detection of recurrences, but no survival benefit.
Abate	History and physical examination, complete blood count, serum chemistry tests, CEA level, and contrast-enhanced CT scan of the chest and abdomen every 3 months for the first 3 years, every 6 months for the next 2 years, and yearly thereafter. PET scans were done yearly.	Median 18 months	Locoregional recurrence - tumor at the anastomotic site or in the lymph nodes in the neck, mediastinum, or upper abdomen. Systemic recurrence -hematogenous metastases to the brain, bone, or visceral organs, or peritoneal carcinomatosis.	29 in symptomatic cohort 145 in asymptomatic cohort	Median overall survival was 20 months.	Frequent early follow-up is appropriate after esophagectomy for adenocarcinoma because 90% of recurrences will occur by 3 years after esophagectomy alone and by 2 years following neoadjuvant therapy. Beyond these time periods, 2% to 3% of recurrences were detected each year, suggesting that annual follow-up is adequate.
Antonowicz	Intensive: history and physical Examination at 6 weeks, 3 months, 6 months, 1 year, 18 months, 2 years, then annually to 5 years. CT scanning at 6 months, 1 year, then annually to 5 years. Regular: unplanned CT following urgent referral due to symptoms	Minimum 37 months	Diagnosed radiologically with or without histology; all recurrent patients had a positive CT scan	17 in the regular surveillance. 44 in unplanned recurrence cohort.	Overall survival: 15 months if regular CT vs 20 months if unplanned CT due to symptoms	Regular surveillance imaging does not influence survival after esophagectomy.
Jiang	Bloodwork, imaging, or EGDs were performed at the discretion of treating physicians. Intensive: surveillance interval of ≤4 months with respect to imaging at any time during the surveillance period Non-intensive: imaging interval > 4 months	Median 38.3 months		53% detected in surveillance 46% detected due to symptoms	Overall survival: 36.2 months in surveillance vs 23.7 months in routine Post-recurrence survival 16.5 months in surveillance vs 4.6 months in routine	Overall survival was longer in surveillance-detected compared to symptomatic recurrences.
Lou	History, physical examination, and chest and abdominal CT scan every 4–6 months for the first 2 years after surgery and then yearly thereafter. Endoscopy every 6 months for 2 years and then yearly thereafter	Median 31 months	Pathologic confirmation or by findings by other study modalities that led to changes in treatment	435 recurrences (38%); 217 detected due to symptoms and 200 due to routine surveillance investigations.	Overall survival was 11 months, and was longer for patients with asymptomatic recurrence.	CT scans of the chest and abdomen, on the other hand, were effective at identifying subclinical recurrences, and its frequency should be based on clinical staging. Surveillance endoscopy has limited value for detection of asymptomatic local recurrence.
Elliott	Intensive surveillance: routine annual CT/PET-CT along with clinical assessment during the first three postoperative years, Standard: investigation as clinically indicated	Median 60 months	—	Intensive surveillance led to reduced symptomatic recurrence (odds ratio 0.17 [0.12–0.25]).	Intensive surveillance improved OS (HR 0.90) and 5-year OS 47.9 ± 1.2% versus 43.2 ± 1.1%.	Intensive surveillance may improve oncologic outcomes, particularly in patients with early stage disease at presentation or with a favorable pathological stage post induction therapy.
DeSouza	CT scanning every 3-months for the first year, every 6-months for the 2nd year, then annually from the 3rd year forward until the 5th year postoperatively	24 months	—	Overall recurrence rate 32%, but 59% detected due to surveillance imaging.		Findings favor the practice of more frequent CT surveillance in the first 2 years, followed by annual surveillance.

### Detection of recurrence rate

**Fig. 2:**
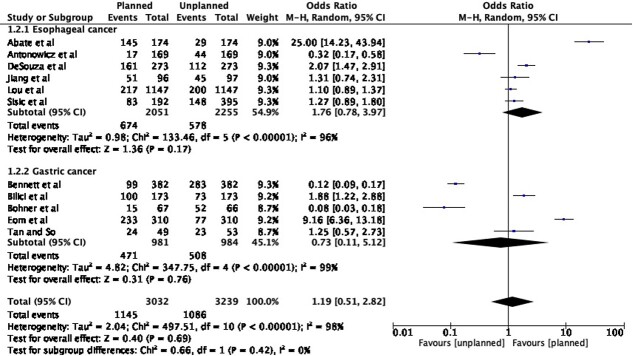
Detection of recurrence in patients undergoing post-operative surveillance for esophagogastric cancer.

In total, 11 studies included a comparison of detection rate of recurrence rate between planned and unplanned surveillance strategies. The total sample size was 3032 patients and 3239 patients in the planned and unplanned surveillance cohorts, respectively. The prevalence of recurrence detected was 1145 and 1086 events in the respective groups. Of these, 674 and 578 patients were detected in planned and unplanned surveillance after surgery for esophageal cancer, while 981 and 984 patients were detected in the respective surveillance patterns after surgery for gastric cancer. Random-effects analysis demonstrated that detection of recurrence was higher with planned surveillance for esophageal cancer, although this trend did not reach statistical significance (OR 1.76, 95% CI 0.78–3.97, *p* = 0.17). Furthermore, no significant difference was seen with planned surveillance for gastric cancers (OR 0.73, 95% CI 0.11–5.12, *p* = 0.76) or when combining the groups (pooled OR = 1.19; 95% CI –0.51–2.82, *p* = 0.69) (Fig. 2).

### Post-recurrence survival

**Fig. 3:**
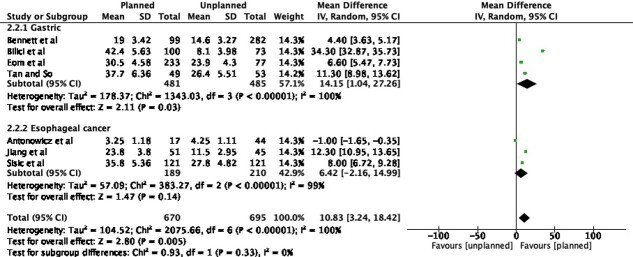
Post-recurrence survival in patients undergoing post-operative surveillance for esophagogastric cancer.

In total, seven studies included a comparison of post-recurrence survival between planned and unplanned surveillance strategies. The total sample size was 670 patients and 695 patients in the planned and unplanned surveillance cohorts, respectively. Data were available for 481 and 485 patients in the respective cohorts for gastric cancer, and 189 and 210 patients in the planned and unplanned surveillance cohorts post-surgery for esophageal cancer. The overall standardized mean difference was 10.83 months (95% CI 3.24–18.42, *p* = 0.005), indicating an overall statistically significant effect. On subgroup analysis, the standardized mean difference for patients who underwent surveillance for esophageal cancer was statistically significant at 14.15 months (95% CI 1.40–27.26, *p* = 0.03), while it was not statistically significant for gastric cancer (SMD 6.42 months, 95% CI –2.16–18.42, *p* = 0.14) (Figure 3). Eight studies also reported on the overall survival between planned and unplanned surveillance. Three of the studies reported a better overall survival with planned surveillance.^23^^,^^28^^,^^30^ Five studies reported no advantage in overall survival. For example, Antonowicz *et al*. reported no overall survival advantage with planned surveillance. In contrast, Abate *et al*. demonstrated that the grouped overall survival was significantly longer in patients treated for the recurrence, although it is difficult to fully attribute this to surveillance strategy, as they had combined both cohorts in survival analysis. For these reasons, a meta-analysis could not be performed as not all the required data for pooled measures was available.^6^^,^^12^^,^^19^^,^^26^^,^^27^

## DISCUSSION

Our study is an updated review of the surveillance programs available for patients who have undergone surgery for gastric and esophageal cancers. Currently, there are no published or ongoing randomized trials comparing intensive surveillance for asymptomatic recurrence with symptom-driven follow-up for esophago-gastric cancers. Thus, we have only identified observational evidence to answer this question. Furthermore, the latest study on this topic was published in 2016, indicating that while there has been much debate around how surveillance should be performed, there has been minimal work dedicated towards it. Based on the studies included in the quantitative analysis, there is no concrete evidence to show that planned surveillance improved the detection of recurrence post-esophagectomy, although post-recurrence survival was improved. In contrast, these effects were not observed for planned surveillance post-gastrectomy. These results, whilst no different to earlier summaries on this topic, emphasize the need for more rigorous evidence in this area.

The main argument for intensive surveillance is due to the high recurrence rate of the cancers. Detection of recurrence at an earlier potentially treatable stage before symptoms arise provides a longer time frame for intervention and may improve survival outcomes. The included studies that carried out routine surveillance reported a high detection rate of recurrence amongst asymptomatic patients, the highest being 75% by Eom *et al*.^27^ However, the impact on survival outcomes is mixed. Jiang *et al*. reported that patients who underwent intensive surveillance had similar pathological characteristics and peri-operative treatments but had similar post-recurrence and overall survival.^23^ In another study, Sisic *et al*. reported that survival analysis of 242 propensity-matched patients showed significantly improved overall survival by 100% for patients with standardized follow-up, and further multivariate analysis showed intensive surveillance to be a positive prognosticator.^21^ In contrast, other studies carried out in Asia, Canada and Europe have reported no clear survival benefit.^24^^,^^26^ It is unclear why detection of recurrence did not translate to a survival advantage in these studies, although this may partly be due to a lack of effective and potentially curative treatment for recurrence, or due to poor study design. Since these studies, there has been a significant improvement in surgical and non-surgical treatments for recurrence, and it is therefore appropriate to revisit whether there may be an improvement in survival outcomes.^32^

Traditionally, the aggressive nature of these cancers deemed even primary treatment not feasible in most patients. Consequently, in the subset of patients who undergo treatment but experience recurrence, further curative treatments were scarcely undertaken.^33^ This led to a lack of surveillance post-operatively, as detection of recurrence often did not merit re-intervention.^20^ However, in recent years, there has been a shift towards curative management of recurrent cancer, at least in cases with loco-regional recurrence. Studies on salvage esophagectomy and chemoradiotherapy have shown promise in providing acceptable survival benefits in selected patients.^34^^,^^35^ The presence of salvage treatments justifies an intensive surveillance approach.^36^ In our paper, majority of the included studies have shown a better overall and post-recurrence survival in patients who underwent intensive surveillance, despite a similar median time to recurrence to account for any lead-time bias. For example, Bennett *et al*. reported longer PRS and disease-specific survival at 13.5 and 29.4 months in patients who underwent intensive surveillance compared to 4.8 months and 21.6 months in the routine surveillance cohorts, respectively.^22^ However, other studies noted no difference in any survival advantage between these surveillance strategies.^20^ One limitation is that not all studies report a clear timeline in time to recurrence and length of follow-up, which may introduce lead-time bias as noted by some studies.^6^^,^^12^ This further necessitates a randomized controlled trial to evaluate all possible metrics of survival in an unbiased manner between routine and intensive surveillance.

In our quantitative analysis, planned surveillance was beneficial for esophageal cancer but not gastric cancer. This dissonance may be explained by better multi-modal treatments available for esophageal cancer that can be used to manage recurrence once detected. For example, in all the included studies, the majority of the patients with recurrence of esophageal cancer underwent either chemotherapy or radiotherapy, whereas not all studies reported patients with recurrence with gastric cancer undergoing these therapies. Furthermore, the sample sizes for studies involving patients with esophageal cancers are larger and therefore their results have more weight towards estimating the overall effect. Moreover, it is possible that surveillance is more rigorous for esophageal cancer. The preliminary results reported by Elliot et al exemplifies the afore-mentioned reasons, since it is a large study involving 4682 patients; 60% of these patients received chemoradiotherapy; and all patients underwent rigorous planned surveillance. Further work is necessary to identify the reasons driving this difference in outcomes.

There are currently no standardized guidelines for monitoring post-operative patients, and a geographical variation is noted in the extent of surveillance. There is unanimity amongst Western nations in advocating follow-up with symptom-driven recurrence investigations only. In Europe, the National Institute for Health and Care Excellence (NICE) and other joint society guidelines advise against routine follow-up (for the detection of recurrence) of asymptomatic patients.^37^^,^^38^ Similarly, the National Comprehensive Cancer Network (NCCN) does not support the use of routine investigations.^39^ The Japan Gastric Cancer Association recognizes that follow-up at outpatient clinic can help patients readjust to normal life and cope with post-gastrectomy syndrome, but addresses the lack of high grade evidence for detection of recurrence and calls for further work ‘to scientifically verify the prognostic relevance of postoperative follow-up programs’.^40^ Hence, while there is consensus that patients should be followed up for a maximum of 5 years and any surveillance should be tailored to the patient’s risk, there is a lack of agreement on how this should be carried out or what it entails, and should form the basis of future work.

Organizational factors have to be considered in implementing any form of surveillance programs. For example, in public healthcare systems such as the National Health Service (NHS), routine surveillance for asymptomatic patients may potentially place undue pressure on secondary and tertiary healthcare sectors. This necessitates the inclusion of general practitioners as well as specific staff and pathways dedicated to looking after this subgroup of patients. In one study, primary healthcare practitioners reported that they would be willing to assume exclusive responsibility for the follow-up care of adult cancer survivors if they were appropriately supported by the specialist center with patient-specific letters from the specialist, printed guidelines, clear routes of rereferral and rapid access to investigations for suspected recurrence.^41^ This is increasingly required in low income countries where cancer is an increasing burden but has gone unnoticed and under-reported due to a lack of nationwide cancer surveillance networks.^42^

### Limitations

Our review is a comprehensive update of existing literature on the surveillance protocols that have been trialed for post-gastrectomy patients. However, there are several limitations that need to be addressed. Firstly, there is no standardized intensive surveillance protocol that has been compared to routine symptom-triggered surveillance, which reduces the comparability of studies to each other. Secondly, the included studies are largely observational in design and have small sample sizes that have often rendered them underpowered. Thirdly, surveillance is largely aimed at identifying recurrence, which depends on patient, surgical and pathological factors; however, most included studies have not tailored their surveillance strategy to factor these variables. Some of the studies have different outcome measures over a variable follow-up time period. Lastly, there is an inherent confounding by indication in studies that have analyzed only patients with recurrence and not comparing it to a more representative cohort of patients without recurrence.

### Areas of future work

Future work should be in the form of well-powered randomized controlled trials that allocate patients to a standardized intensive surveillance program; a routine surveillance program based on symptoms; or no further surveillance. Patients should be stratified based on intraoperative factors, oncological factors and pathological factors, and need for alternative multimodal treatments to fully account for any other confounding variables. The survival outcome measures attained over an adequately long follow-up time would better inform whether intensive routine surveillance offers any viable advantage. Ultimately, the feasibility of surveillance depends on the infrastructure set up to facilitate it, and this varies with the structure of the healthcare system.^42–44^ Economic analysis tailored to organizational factors have to be incorporated to conclude if routine intensive surveillance is feasible.

## CONCLUSION

Gastric and esophageal cancers are malignancies associated with a poor prognosis, largely due to their high recurrence rates. Yet, there is no standardization of guidelines internationally to inform clinicians of how patients should be monitored after their operation. Although this has been a topic of debate for decades, our study shows that there is still no consensus on this. More importantly, our study highlights that planned surveillance has good survival benefit for patients with esophageal cancer, while there is a lack of high grade evidence for its benefit in gastric cancers. Further work in the form of randomized controlled trials is required to confirm these findings and subsequently establish how surveillance should be carried out and wand what it should comprise of in a practical and clinically safe manner.
